# Runyan's Method of Bridge-Work

**Published:** 1887-04

**Authors:** H. W. Runyan

**Affiliations:** Eaton, Ohio.


ARTICLE VII.

### RUNYAN'S METHOD OF BRIDGE-WORK.

BY H. W. RUNYAN, D. D. S., EATON, OHIO.

There is no doubt that bridge-work is very valuable in many instances for partial dentures. But the great cost of the gold process places it within the reach of comparatively few, while there are fewer practitioners of dentistry that thoroughly understand the swaging and soldering of gold that is necessary in the construction of the gold bridge-work. The method here described will place it within the reach of all who can afford a plate of any kind, and it can be constructed by any one capable of making a vulcanite plate, and I think it will last as long as any bridge-work, or as long as the roots, to which it is attached, will last.

*Process of Construction:* For a case where the four incisors are missing and the cuspid roots remain:—

After cutting the cuspids down to, or a little above, the margin of the gum, prepare by drilling out the canal with an inverted cone bur, and then a pointed fissure bur. By so doing, a perfect funnel-shaped canal is formed, which gives strength to the work, and facilitates access to the end of the root. Take a platinum bar long enough to reach from one root to another, and bend at right angles to form the pins. Now set the bridge support in place, after bending to conform with the gums; and take the impression and articulation. Make the model, place on the articulator and wax on vulcanite teeth. Remove from the articulator, flask and vulcanize, after covering all the rubber with vulcanizable gold.

Gum teeth can be used for the bridge between the roots, if the alveolar process has been absorbed very much.

After vulcanizing, clean up and fasten in by placing a little cement on the pin that extends into the cavity formed by the fissure drill. The rubber will fill that part formed by the inverted cone.

Use the best rubber, run the vulcanizer up slowly to 300° Fah., and vulcanize for one hour and fifteen minutes. You will have "a thing of beauty, and a joy" to your patient and yourself.—*Ohio Journal of Dental Science.*

